# Visualization of Tumor-Immune Interaction - Target-Specific Imaging of S100A8/A9 Reveals Pre-Metastatic Niche Establishment

**DOI:** 10.7150/thno.17138

**Published:** 2017-06-15

**Authors:** Michel Eisenblaetter, Fabian Flores-Borja, Jae Jin Lee, Christina Wefers, Hannah Smith, Rebekka Hueting, Margaret S Cooper, Philip J Blower, Dominic Patel, Manuel Rodriguez-Justo, Hanna Milewicz, Thomas Vogl, Johannes Roth, Andrew Tutt, Tobias Schaeffter, Tony Ng

**Affiliations:** 1 Richard Dimbleby Department of Cancer Research, Randall Division & Division of Cancer Studies, King's College London, London SE1 1UL, UK; 2 Division of Imaging Sciences & Biomedical Engineering, King's College London, London SE1 7EH, UK; 3 Department of Clinical Radiology, University Hospital Muenster, 48149 Muenster, Germany; 4 Breast Cancer Now Research Unit, Department of Research Oncology, Guy's Hospital, King's College London, London SE1 9RT, UK; 5 Department of Histopathology, University College London, London WC1; 6 UCL Cancer Institute, Paul O'Gorman Building, University College London, London WC1E 6DD, UK; 7 Institute of Immunology, University Hospital Muenster, 48149 Muenster, Germany

**Keywords:** Premetastatic niche, tumour immunology, imaging, MDSC.

## Abstract

*Background *Systemic cancer spread is preceded by the establishment of a permissive microenvironment in the target tissue of metastasis - the premetastatic niche. As crucial players in establishment of the pre-metastatic niche, myeloid derived suppressor cells (MDSC) release S100A8/A9, an exosomal protein that contributes to metastasis, angiogenesis, and immune suppression. We report the application of antibody-based single-photon emission computed tomography (SPECT) for detection of S100A8/A9 *in vivo* as an imaging marker for pre-metastatic tissue priming.

*Methods *A syngeneic model system for invasive breast cancer with (4T1.2) or without (67NR) the tendency to form lung metastasis was established in BALB/c mice. A SPECT-probe has been generated and tested for visualization of S100A9 release. Tumor-associated changes in numbers and fuction of immune cells in pre-metastatic tissue were evaluated by flow cytometry and confocal microscopy.

*Results *S100A8/A9 imaging reflected MDSC abundance and the establishment of an immunosuppressive environment in pre-metastatic lung tissue (activity 4T1.2 vs. healthy control: 0.95 vs. 0.45 %ID; p<0.001). The S100A8/A9 imaging signal in the pre-metastatic lung correlated with the subsequent metastatic tumor burden in the same organ (r^2^=0.788; p<0.0001). CCL2 blockade and the consecutive inhibition of premetastatic niche establishment was clearly depicted by S100A9-SPECT (lung activity untreated vs. treated: 2 vs, 1.4 %ID).

*Conclusion *We report S100A8/A9 as a potent imaging biomarker for tumor-mediated immune remodeling with potential applications in basic research and clinical oncology.

## Introduction

The induction of a tumor-permissive cellular environment in target tissue of metastasis is driven by tumor secreted factors, which can modify the local immune effector cell function at sites distal to primary tumor and prior to the engraftment of circulating tumor cells 1, 2. The pre-metastatic niche is characterized by stromal reorganization and the infiltration of immune cells including CD11b^+^ myeloid-derived suppressor cells (MDSC) 3 4 which induce expansion and local accumulation of regulatory T cells (Treg) and suppress the anti-tumor immune response of NK cells 5. MDSC accumulate in the primary tumor in response to a variety of cytokines and soluble factors including granulocyte colony stimulatory factor (G-CSF) and TGFβ, released by tumor and/or non-tumor cells 6. MDSC accumulation in the primary tumour and recruitment to pre-metastatic lungs 7 is mediated by the protein heterodimer S100A8/A9 8, members of the S100 protein family. MDSC express receptors for S100A8/A9 and initiate an autocrine loop to increase local levels of the heterodimer either by direct secretion into the interstitium or as a major component of exosomes 9. S100A8/A9, a ligand for pattern recognition receptors RAGE and TLR4, induces the production of pro-inflammatory mediators such as serum amyloid A (SAA) 3 in the pre-metastatic lungs, to attract further CD11b^+^ MDSC 10, 11. Generally, high S100A8/A9 levels in tumor tissue or serum indicate a more rapid, aggressive course of disease 12, 13 in various human cancers. Non-invasive detection and measurement of exosomal S100A8/A9 release in potential pre-metastatic sites would strongly promote the clinical utility of this marker.

We recently established target-specific *in vivo *S100A8/A9 optical imaging for monitoring of monocyte activity in local inflammation 14 and primary cancer lesions 15, using fluorescence-labeled antibodies. The local S100A8/A9-release correlated with the accumulation of tumour-associated CD11b^+^ cells and proved predictive of tumor development. To enable systemic S100A9 imaging and explore the potential of S100A9 as a translatable diagnostic marker, we have now developed S100A9-specific single photon emission computed tomography (SPECT) imaging. Using a murine model of metastatic breast cancer, we present the first *in vivo* visualization of systemic tumor-mediated effects on immune cells and establish S100A8/A9 as a surrogate marker for immunomodulation in the context of pre-metastatic lung tissue priming. Whole-body S100A8/A9 SPECT imaging mirrored the establishment of a metastasis-permissive microenvironment and allowed for assessment of the immunosuppressive state in pre-metastatic lung tissue.

As PN establishment also includes endothelial activation, several factors associated with vascular reorganization have been exploited as indicators of premetastatic processes, including VEGFR and avb3 16, 17. The possibility to intervene in the metastatic process by blockade of endothelial activation and consecutive cell adhesion has been assessed 18, suggesting these markers might serve as valuable indicators of PN formation. However, these processes are only consecutive to tumor-mediated immune reprogramming and activation in distant tissue and therefore dependent on CCL2/CCR2 driven S100A8/A9 release.

## Results

### *In vivo* imaging of S100A8/A9 distribution by preclinical SPECT-CT

**Figure 1:**
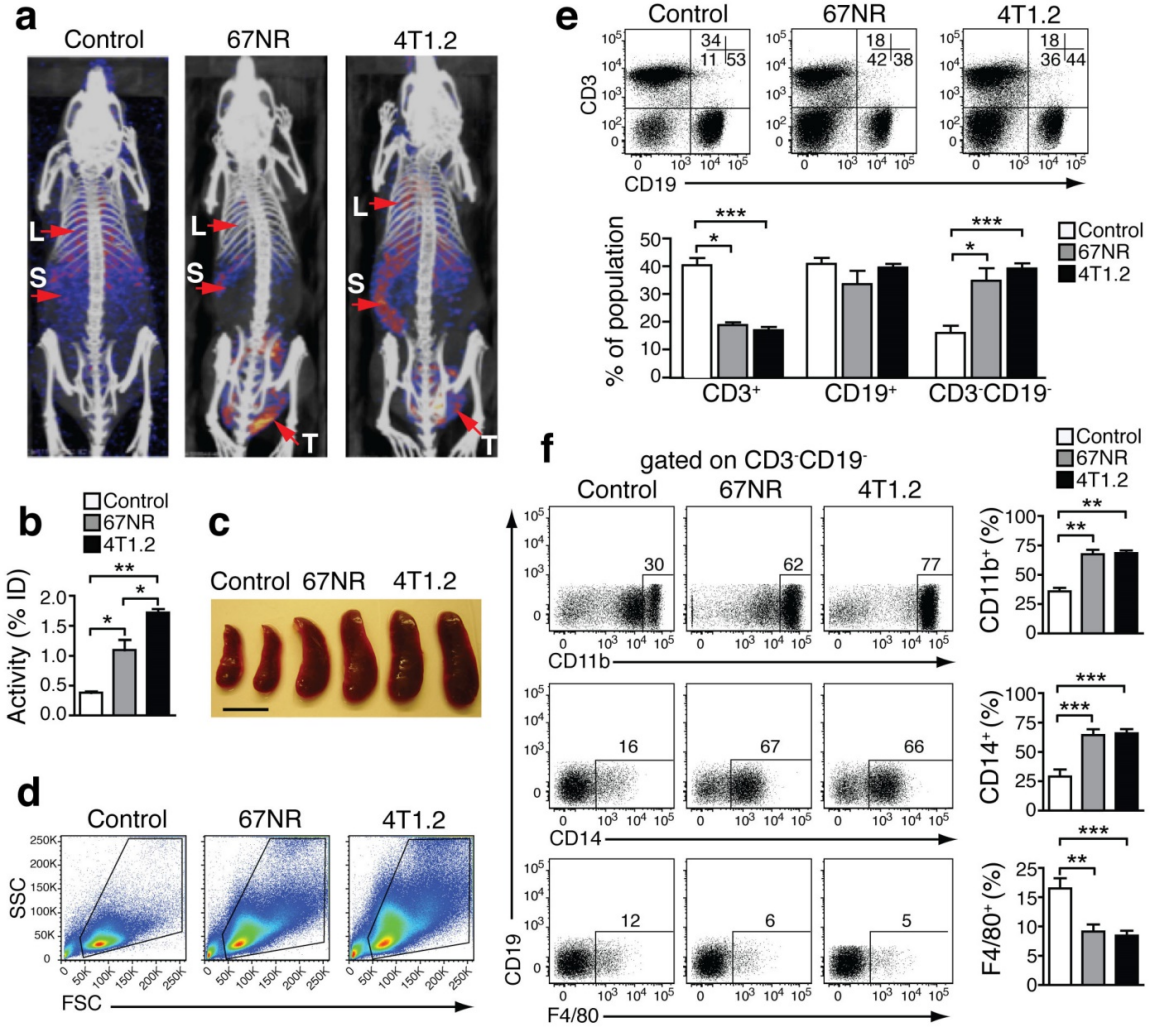
** Increased S100A8/A9 and monocytes in tumor-bearing mice.** (**a**) S100A9-SPECT imaging at day 21 after tumor induction. Specific tracer uptake in lungs (L) and spleen (S) of tumor-bearing animals can be detected, reflecting the grade of malignancy (4T1.2>67NR). (**b**) Corresponding activity graph showing the relative tracer *in vivo* uptake in the spleen. (**c**) Representative images showing splenomegaly in 67NR and 4T1.2 tumor-bearing mice. Scale bar= 0.5 cm. (**d**) Representative FSC vs. SSC FACS plots showing changes in the distribution of spleen cells in tumor-bearing mice as compared to healthy control mice. (**e**) FACS plots showing frequency of CD3^-^CD19^-^ gated on spleen live cells in tumor-bearing mice. Bar graphs show the cumulative data for mean percentage of CD3^+^CD19^-^, CD3^-^CD19^+^ and CD3^-^CD19^-^ cells. (**f**) Expression of monocytes/macrophage markers CD11b (top), CD14 (middle) and F4/80 (bottom) in CD3^-^CD19^-^ spleen cells. Bar graphs show the mean percentage of each monocyte marker in the CD3^-^CD19^-^ cell population. (**c**-**f**) All non-tumor-bearing control mice (n=14), 67NR (n=9) and 4T1.2 (n=16) tumor-bearing mice samples were analysed 14d after tumor induction in three independent experiments. Mean ± SE: ***p<0.005, **p<0.01, *p<0.05.

We performed SPECT imaging using an S100A9-specific antibody labeled with Indium-111 (In-111) in 4T1.2- and 67NR-tumor-bearing mice 19-21. 4T1.2 tumors form metastasis in lungs and bones while 67NR tumors grow without shedding cells systemically. S100A9-specific SPECT imaging showed a higher tracer uptake in lungs, spleen and tumor of mice, implanted with metastatic 4T1.2, as compared to non-metastatic 67NR and non-tumor-bearing control mice (Figs 1a and 1b). Estimation of nonspecific tracer distribution, using an Indium-labelled rabbit antibody of irrelevant specificity, showed no differences between non-tumor bearing controls and the two tumor entities. The increased S100A9 signals in the spleen of 4T1.2 and 67NR tumor-bearing animals (Fig 1b) were accompanied by splenomegaly (Fig 1c) and changes in the cellular composition of the splenic cell populations (Fig 1d, e). We observed a significant increase in the frequency of CD3^-^CD19^-^CD11b^+^CD14^+^monocytes (Fig 1f) in 67NR and 4T1.2 tumor-bearing mice. Monocytes in tumor-bearing mice were on average less differentiated as judged by the decreased frequency of F4/80^+^ cells (Fig 1f, lower panel) and CD80 expression in CD3^-^CD19^-^ splenocytes (Supplementary Fig 1) as compared to the respective controls.

### A specific population of pro-inflammatory monocytes is increased in mice with higher metastatic burden

**Figure 2:**
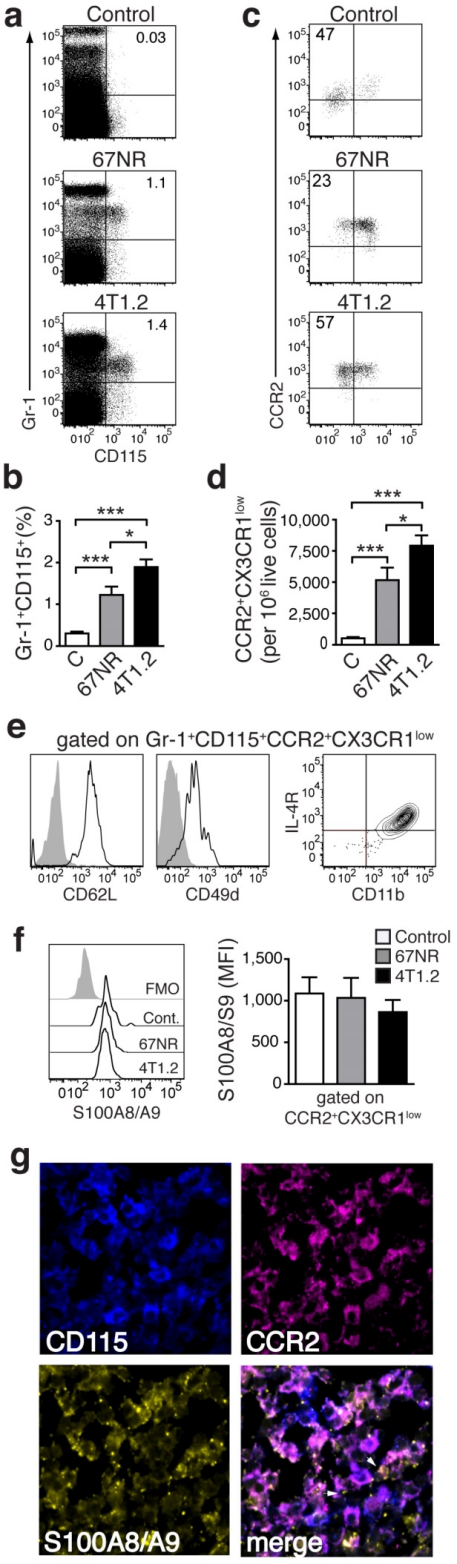
** Monocytes in tumor-bearing mice share markers with MDSC and express S100A8/A9. **(**a**,** b**) Representative plots of splenocytes gated on live cells showing the frequency of Gr-1^+^CD115^+^ cells in control non tumor-bearing mice, 67NR and 4T1.2 tumor-bearing mice. The bar graph shows results from 6 independent experiments. (**c**,** d**) Representative plots of monocytes gated on Gr-1^+^CD115^+^ showing the frequency of CCR2^+^CX3CR1^low^ cells in control non tumor-bearing mice, and 67NR and 4T1.2 tumor-bearing mice. The bar graph shows average relative number of CCR2^+^CX3CR1^low^ per 10^6^ live cells from 6 independent experiments. (**e**) Expression of CD62L, CD49d, IL-4R and CD11b in CCR2^+^CX3CR1^low^ in splenocytes derived from 4T1.2 tumor-bearing mice (n=4). (**f**) Expression of intracellular S100A8/A9 in CCR2^+^CX3CR1^low^ monocytes in samples described in panels **a**-**d**. (**g**) Representative confocal microscopy images showing expression of CD115 (blue), CCR2 (magenta) and S100A8/A9 (yellow) in frozen spleen sections from 4T1.2 tumor bearing mice (n=3); scale bar= 10μm. A rabbit anti-S100A9 antibody was used for staining and this is indicative of the extracellular location (white arrows) of the dimer as A8/A9 are only secreted as a heterodimer. Results in panels **a-d**,** f**, are from non tumor-bearing control mice (n=15), 67NR (n=16) and 4T1.2 (n=14) tumor-bearing mice samples were analysed 14d after tumor induction in three independent experiments. Mean ± SE: *** *p*<0.005, **p<0.01, *p<0.05.

Analysis of the cellular infiltrate in spleens from tumor-bearing animals in comparison to control mice allowed for definition of subset of monocytes (CD3^-^CD19^-^CD11b^+^CD14^+^) (Fig. 1f), increased in 4T1.2 tumor-bearing animals as compared to 67NR. This provided an immunological correlate for the differences in *in vivo* imaging between the two models (Figure 2 a-c). The increased monocyte population was Gr-1^+^CD115^+^ (Fig 2a, b) and expressed a phenotype, CCR2^high^CX3CR1^low^, defining pro-inflammatory monocytes 22 (Figure 2c, d). These cells also expressed CD62L, CD49d, CD11b and interleukin-4 (IL-4) receptor (Fig 1e), suggesting them as a subgroup of MDSCs 23. We could moreover show this subpopulation to be positive for S100A8/A9 (Fig 2f). Confocal microscopy of frozen spleen sections showed intracellular and extracellular S100A8/A9 in CD115^+^CCR2^+^ cell clusters (extracellular S100A8/A9 aggregates indicated by white arrows in Fig 2g).

### Release of S100A8/A9 by Gr-1^+^CD115^+^CCR2^high^CX3CR1^low^monocytes is regulated via the CCL2/CCR2 pathway

**Figure 3:**
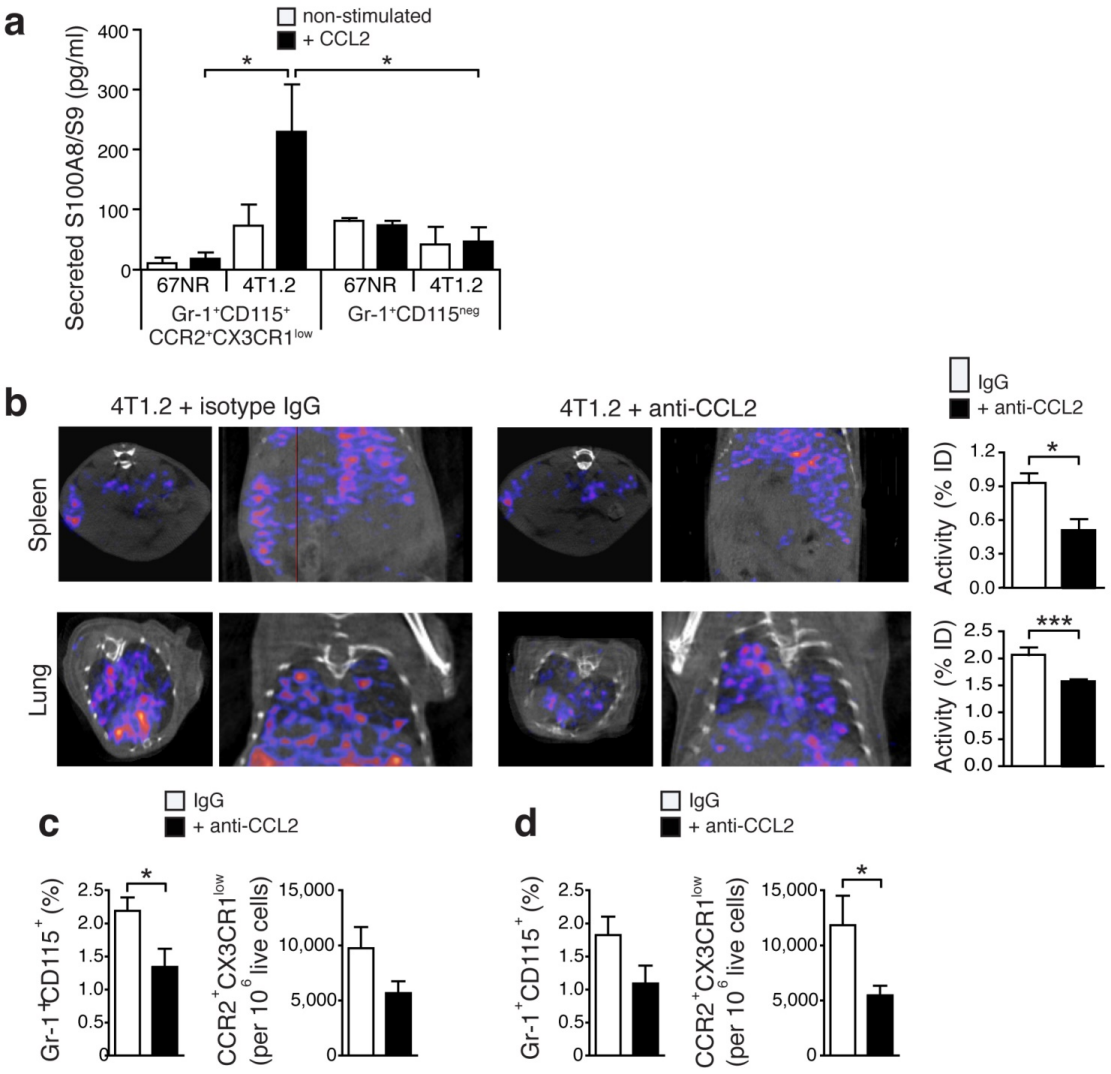
** Secretion of S100A8/A9 by pro-inflammatory monocytes is under control of CCL2. **(**a**) S100A8/A9 concentration in supernatant of FACS-sorted CCR2^+^CX3CR1^low^ pro-inflammatory monocytes (n=5) and Gr-1^+^CD115^neg^ (n=4) from spleens of 67NR and 4T1.2 tumor-bearing mice stimulated 48h with recombinant mouse CCL2. S100A8/A9 concentration was determined by ELISA in two independent experiments. (**b**) Axial and coronal images from SPECT analysis and relative *in vivo* tracer uptake in 4T1.2 tumor-bearing mice treated with an isotype control antibody (IgG) or a blocking anti-CCL2 antibody. The frequency of CCR2^+^CX3CR1^low^ and average relative number of CCR2^+^CX3CR1^low^/10^6^ live cells in spleens (**c**) and lungs (**d**) (n=4, for each organ and treatment analysed in two independent experiments 14d after tumor induction) is reduced under CCL2-blockade. Mean ± SE: *p<0.05.

The CCR2-CCL2 signaling axis has been implicated in breast cancer cell seeding in the lungs 24. Upon *in vitro* stimulation with recombinant CCL2, monocytes (Gr-1^+^CD115^+^CCR2^high^CX3CR1^low^) derived from spleens of 4T1.2-bearing mice secreted a significantly higher amount of S100A8/A9 as compared to cells from 67NR-bearing mice or matched samples of granulocytes (Gr-1^+^CR2^+^CD115^-^) (Fig 3a). Considering earlier reports of CCL2 as a chemo-attractant for MDSC 25, we assessed whether blocking this chemokine would affect the release of S100A8/A9, reflected by *in vivo *SPECT imaging, and the consecutive recruitment of Gr-1^+^CD115^+^CCR2^high^CX3CR1^low^monocytes to pre-metastatic lung tissue. CCL2-blocking with neutralizing antibodies led to a significant reduction of the S100A9 signal in spleens and lungs of 4T1.2-bearing animals (Fig 3b). *Ex vivo* FACS analyses revealed a significant reduction of Gr1^+^CD115^+^CCR2^high^CX3CR1^low^ monocytes in respective tissue samples (Figs. 3c and 3d).

### Pro-inflammatory monocytes in lung tissue promote an immune environment that favors metastasis

**Figure 4:**
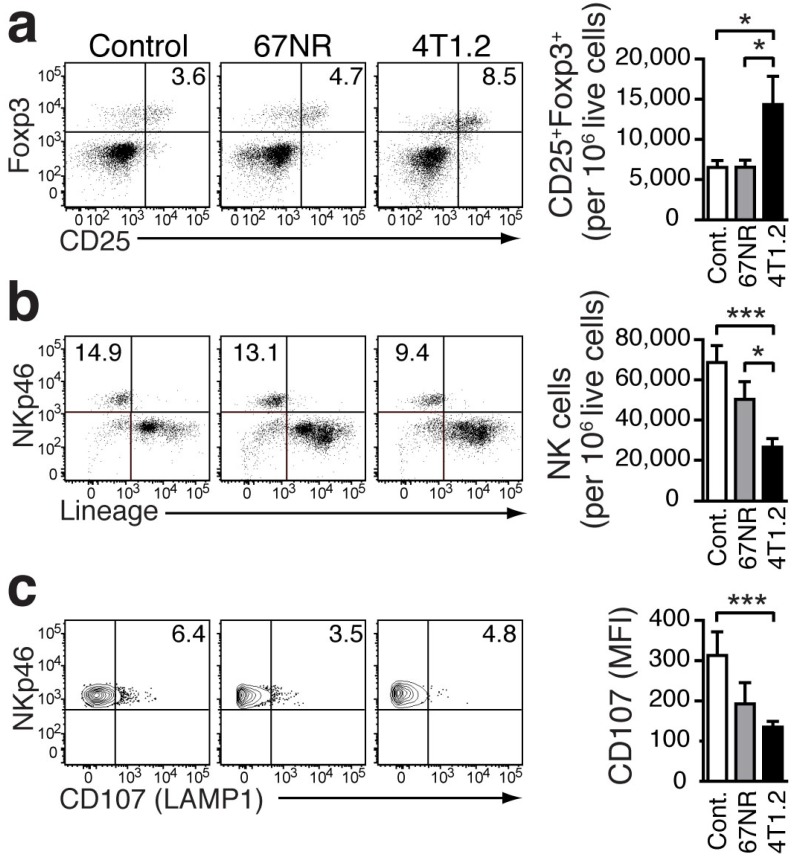
** Immunosuppressive environment induced in the lungs of 4T1.2 tumor-bearing mice. **Representative FACS plots and bar charts showing the frequency of Tregs (**a**), NK cells (**b**), and CD107^+^ cells (**c**) in the lungs of non-tumor bearing control mice (n=9, **a, b**; n=7, **c**), 67NR (n=11, **a, b**; n=6, **c**), and 4T1.2 tumor-bearing mice (n=11, **a, b**; n=6, **c**). Results were obtained in three independent experiments. For gating strategy, see Supplementary Figure 2. Mean ± SE: *** *p*<0.005, *p<0.05.

In our breast cancer models, the increased frequency of Gr-1^+^CD115^+^CCR2^high^CX3CR1^low^monocytes in the lungs of tumor-bearing mice was associated with an increased presence of CD25^+^Foxp3^+^Treg (Fig 4a). Compared to healthy controls, the increase was more pronounced in 4T1.2-bearing mice. Treg control the proliferation and cytotoxic activity of NK cells 26. The increase in Treg in the pre-metastatic lung tissue was concomitant with a decreased number of NK cells (Figure 4b). Although the differentiation of NK cells was not affected (Supplementary Fig 4), their activation, as indicated by staining for CD107 (LAMP1 which measures degranulation function) 27, was impaired in the presence of Treg and Gr-1^+^CD115^+^CCR2^high^CX3CR1^low^monocytes (Fig 4c).

### S100A9-SPECT reflects Gr-1^+^CD115^+^CCR2^high^CX3CR1^low^myeloid cell accumulation in the pre-metastatic lung and predicts consecutive tumor cell seeding

**Figure 5:**
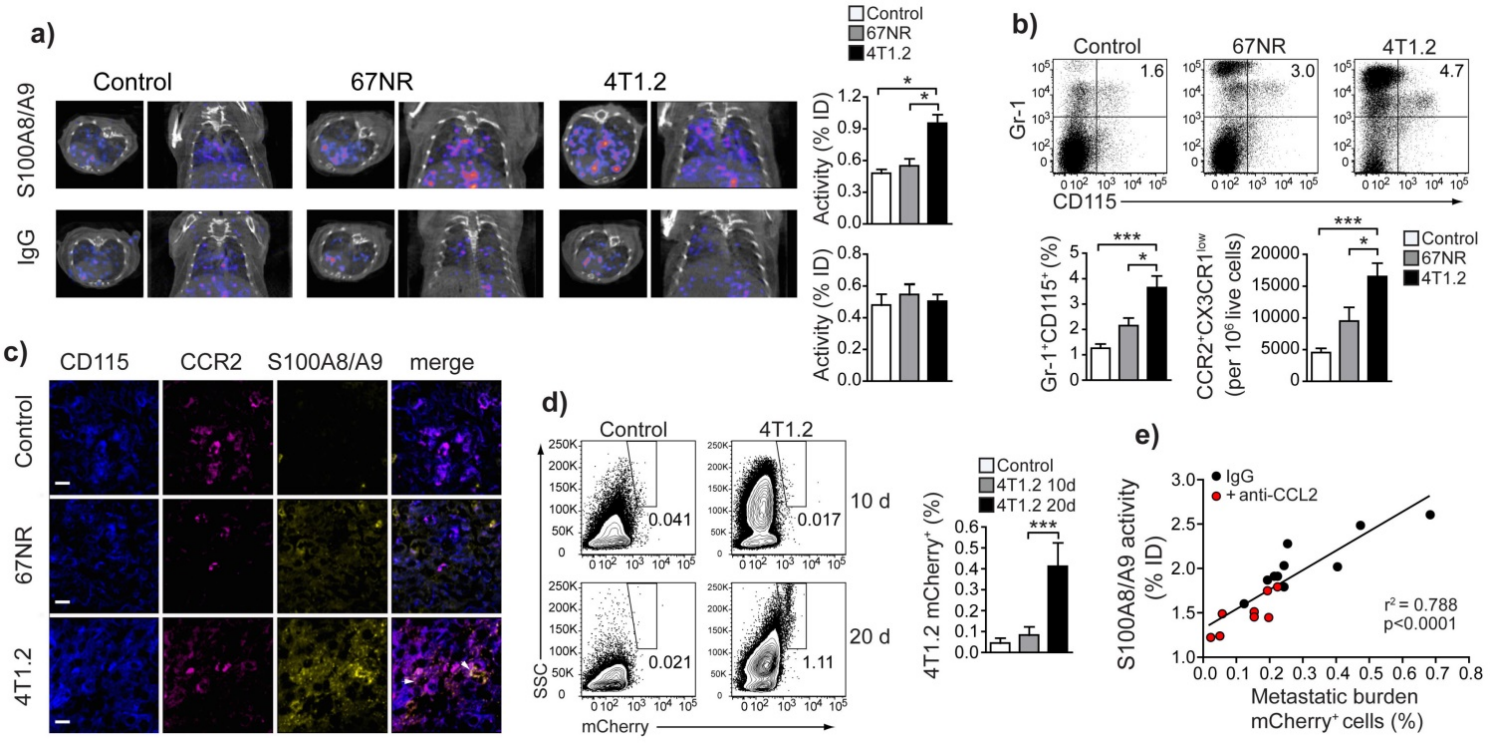
** S100A8/A9-induced immune remodelling in lungs of mice 4T1.2-tumor bearing mice predicts metastasis. **(**a**) Exemplary axial and coronal *in vivo* images from SPECT examination and relative tracer accumulation graphs of healthy control animals and 67NR or 4T1.2 tumor-bearing mice (d10 after tumor induction) after injection of the S100A8/A9-specific tracer or unspecific IgG to control for perfusion effects. While the unspecific IgG does not show any differences between the three groups, S100A9-SPECT reveals ongoing monocytes activation and immune remodelling in the 4T1.2 tumor-bearing mice, reflected by a strong tracer-accumulation. (**b**) Frequency of Gr-1^+^CD115^+^ CCR2^+^CX3CR1^low^ monocytes in lungs of control non tumor-bearing mice, 67NR and 4T1.2 tumor-bearing mice. The bar graph shows average frequency of Gr-1^+^CD115^+^ and the relative number of CCR2^+^CX3CR1^low^/10^6^ live cells from 5 independent experiments. (**c**) Expression of CD115 (blue), CCR2 (magenta) and S100A8/A9 (yellow) in frozen lung sections from 4T1.2-tumor bearing mice (n=3). Extracellular S100 signal is indicated by white arrows. (**d**) Representative plots and bar graph showing the frequency of mCherry^+^ 4T1.2 cells in the lungs of 4T1.2 tumor-bearing mice at 10 and 20 days after tumor induction (n=4, one of two experiments shown). (**e**) Correlation between S100A8/A9 activity in the lungs of 4T1.2 tumor-bearing mice at day 10 and the frequency of mCherry^+^ 4T1.2 tumor cells at day 21 after tumor induction (n=9). Red dots indicate animals that received anti CCL2 treatment. Mean ± SE: *** *p*<0.005, *p<0.05.

The frequency/number of Gr1^+^CD115^+^CCR2^high^CX3CR1^low^monocytes in primary tumor and peripheral blood (Supplementary Fig 3) and *in vivo* S100A9-SPECT signal in lungs (Figure 5a, top panels and Fig 5b) correlated with the respective metastatic potential of the underlying model: inflammatory monocytes and S100A9 were significantly increased in the 4T1.2 model as compared 67NR or control mice. Rabbit IgG (rabIgG) of irrelevant specificity served as a control for perfusion-related and non-specific tracer accumulation. RabIgG-driven SPECT did not show distribution differences between the tumor entities and controls (Fig 5a, bottom panels). Confocal microscopy of frozen lung sections from 4T1.2 tumor-bearing mice revealed increased intra- and extracellular S100A9 in areas of CCR2^+^CD115^+^ monocyte accumulation (Fig 5c), thus confirming the *in vivo *imaging (Figs. 1a and 5a). We found increased S100A8/A9 levels in lungs of 4T1.2-tumor bearing mice as early as ten days after tumor inoculation when no local metastatic deposit of tumor cells was evident. At later time points (20 days), 4T1.2 tumor cells were clearly detectable in equivalent tissue samples (Fig 5d). Increased S100A9-levels at day 10 after tumor implantation were indicative of the tumor-mediated immune remodeling and correlated with the consecutive tumor cell seeding into the lung (Fig 5e). To increase the variability in the homogeneous 4T1.2 model, animals that received anti-CCL2 treatment were included in the correlation and S100A9-SPECT reflected the reduced accumulation of S100A8/A9-releasing cells in the pre-metastatic lung tissue. The consecutive metastatic burden as depicted by tissue analysis at day 20 after tumor inoculation was respectively reduced.

## Discussion

The crosstalk between tumor and immune cells is recognized to be of major importance for tumor spread and development. Current assessment of tumor-mediated immune cell regulation is based on the quantification of different cell subsets in biopsies 28 and diagnostic markers that allow for non-invasive, continuous monitoring of tumor-mediated inflammation and tumor-immune cell crosstalk are elusive. This precludes optimization of and selection for cancer immunotherapy 29 and hampers further understanding of the events, preceding the establishment of metastasis.

This study provides the first evidence that S100A8/A9 can be used as a non-invasive imaging marker, for the establishment of a tumor-permissive, immunosuppressive environment in target organs of metastasis. S100A8/A9 release was evident and detectable at an early stage of the metastatic process before tumor cells seeded in the lungs of 4T1.2 tumor-bearing mice (Figure 5d) and presented along with increased numbers of MDSC-like cells and Treg. (Figs 1, 5). Our results are supported by studies showing that MDSCs mediate the development of tumor-induced Treg 30, required for metastasis 31 and this could explain our observation of decreased number and activity of NK cells (Figs 4b, c)^32^.

The relevance of S100A8/A9 in malignant disease has been highlighted by studies showing that S100-deficient animals present a reduced Gr-1^+^CD11b^+^ MDSCs resulting in a decreased growth of lymphomas and sarcomas 8. The important role for S100A8/A9 in priming organs such as the brain and lung, for metastastic predisposition, has been demonstrated in murine breast cancer models 33. In addition, clinical studies of patients with high grade and invasive breast tumors have revealed S100A8/A9 as marker of poor prognosis 34. Despite the lack of a signal peptide sequence, S100A8/A9 is actively delivered to the tumor microenvironment and distant tissues via exosomes that are secreted by tumor-associated immune cells such as MDSC 7. Upon release, S100A8/A9 further promotes the accumulation of MDSC 9, thereby orchestrating an immunosuppressive microenvironment in pre-metastatic tissue, optimal for tumor cell deposition and growth 10, 33. In agreement with those studies, we demonstrated by *in vivo* SPECT imaging, FACS analyses and histology, increased S100A8/A9 levels in pre-metastatic lung tissue, and a concomitant accumulation of MDSC-like monocytes (Figs 1, 2, 5). The *in vivo* imaging, indicative of tumor-tissue interaction and immune remodeling, proved predictive for subsequent metastatic tumor burden at an individual animal level (Fig 5e).

Previously, markers Gr-1, CD11b and CD115 have been used to identify tumor-induced MDSCs 24, 35. We have further specified this phenotype with the use of additional markers CCR2 and CX3CR1 which allowed us to define a specific population, induced in our tumor model (Fig 2) and to give a hint towards possible immune regulation. The cell type we found increased in accordance with the malignant potential of induced tumors shares surface markers of active, pro-inflammatory monocytes, but lacks markers for mature macrophages. In fact, we find the ratio of immature to mature cells of monocytic origin shifted towards immature phenotypes - in line with reports that tumors promote an arrest of certain cell types in immature stages 36.

In addition to S100 proteins, chemokines and chemokine receptors are among the soluble factors that contribute to lung metastasis in cancer. Hiratsuka *et al.*, have recently shown that primary tumors are able to induce changes in vascular permeability, preparing homing sites for circulating, metastatic tumor cells 37. The CCL2-CCR2 axis seems to be crucial in this process. CCL2, secreted by tumor-associated fibroblasts induces the attraction of monocyte-like cells, the mobilisation of bone marrow-derived myeloid cells 24, 37 and tumor-associated fibroblasts 25, 38. We show that Gr-1^+^CD115^+^CCR2^high^CX3CR1^low^monocytes secrete S100A8/A9 upon stimulation with recombinant CCL2 (Fig 3a). Several different cells including various tumor cells and immune cells have been reported as potential producers and providers of S100A8/A9 and this can also be found in the tumor microenvironment as well as in premetastatic tissue. For the experimental model we have been using, we could however exclude tumor cells as the source of S100A8/A9, by analysis of cell lysates and tissue culture supernatants, and by FACS of harvested tumor tissue, showing virtually all S100A9^+^ cells in the 4T1.2 model, to be CD11b^+ ^15. Metastatic breast cancer is known to secrete CCL2 39, which can stimulate MDSC-like and trigger S100A8/A9 release (Fig. 3a). Exosomal S100A8/A9, released upon CCL2 stimulation in the primary tumor, can be delivered systemically to the pre-metastatic lung endothelium, and potentially stimulate the paracrine production of mediators such as serum amyloid A3 (SAA3), attracting further CD11b^+^ cells to the lung 10. We also found CCL2 positive cells in premetastatic lung tissue, in clusters potentially representing the developing premetastatic miche (PN) with consecutive release of S100A8/A9 and other bookmarking factors. The effects of *in vivo* blocking of CCL2 activity highlighted the importance of this positive cooperativity in our model. Treatment with CCL2 blocking antibodies resulted in reduced S100A8/A9 expression and a significantly decreased number of pro-inflammatory monocytes in both spleen and lungs of 4T1.2-tumor bearing mice (Fig 3b-d). Blocking antibodies to CCL2 such as Carlumab (Johnson & Johnson, New Brunswick, NJ, USA), have been tested in clinical trials and preliminary antitumor activity of this approach in advanced cancer patients was observed 40.

The use of S100A8/A9 as a clinical marker for cancer immune modulation and translation into specific imaging approach will depend in part on further development of the tracer. Since the S100A8/A9 molecular target is extracellular, full-length antibodies are expected to have good access to it. Nevertheless, full-length antibodies are only slowly eliminated from the blood pool, leading to a long period between tracer applications and imaging with reasonable contrast to unspecific background-signal requiring isotopes with a long half-life. Smaller targeting compounds - either antibody fragments or non-peptidic small molecules should in this context be evaluated. A small molecule, binding S100A8/A9, would potentially exhibit a more rapid clearance from the blood pool and faster accumulation in the target tissue, hence enabling for the use of short-lived isotopes for clinical PET imaging. Labeling of such compounds for other imaging modalities for clinical or preclinical use would also be conceivable. Moreover, a correlation with conventional tumor grading and serum markers for tumor development would foster the clinical use of S100A8/A9 as a marker for patient stratification and a prognostic potential as indicated by our results. The signal, obtained *in vivo* using the above mentioned antibody would basically be constituted by both, the biologically active S100A8/A9 heterodimer as well as the less abundant S100A9 monomer 41. The latter has also been assigned pro-inflammatory and pro-migratory functions, thereby potentially contributing to the overall effect of S100A8/A9.

For cancer therapy, experimental cancer research and drug development, an opportunity to follow immune cell activity in a specific way over time will have a significant impact. For instance, despite the success and exciting potential of new therapeutic agents, such as monoclonal anti-PD1 or anti-CTLA-4 blocking-antibodies 29, response varies greatly (reviewed in 42) and non-invasive tools for monitoring of the conditioning of micrometastatic niches are needed to further optimise therapy and to sustain survival benefits in patients. As a paradigm for such future work, we suggest S100A8/A9 *in vivo* imaging as a first approach to non-invasively address tumor immune crosstalk. Clinical establishment of this technique could change how patients are selected for and monitored during therapy.

## Material and Methods

### *In vivo* breast cancer model

1x10^6^ cells of the murine (BALB/c) breast cancer cell lines 4T1.2 and 67NR^19^ were implanted orthotopically into the mammary fat pad of 5-6 week-old female BALB/c mice (Charles River UK, Margate, UK).

Mouse organs were dissected at different time points and cell suspensions prepared for flow cytometry analyses and cell sorting. Sorted monocyte populations were plated in 96-well plates and treated with 50ng/ml recombinant mouse CCL2 (Invitrogen). After 48hrs, supernatants were screened for S100A8 using ELISA (DuoSet, R&D systems) according to manufacturers' instructions.

For some experiments, tissues snap-frozen and 4-5 μm thick sections were cut and stored at -80°C for further immunofluorescence staining and confocal microscopy analysis.

For CCL2 blocking, 4T1.2 tumor-bearing mice received an intraperitoneal injection of 100µg goat polyclonal anti-CCL2 antibody (R&D, Abingdon, UK) every other day beginning on day 4 after tumor inoculation, following established protocols 43. Control mice were treated with 100μg normal goat polyclonal serum. All experimental *in vivo* procedures were covered by project licences, issued by the UK Home Office and conducted according to the Animals Scientific Procedures Welfare Act.

### Tracer synthesis

The protocol for labelling antibodies for SPECT imaging has been described in detail earlier 44. Briefly, a full length polyclonal antibody (1-2 mg/mL), targeting the A9 subunit of the S100A8/A9 heterodimer was incubated with 50 mM EDTA in 0.1 M ammonium acetate for chelation of metal contaminations. The purified antibody was suspended in 0.1 M HEPES buffer, pH 8.5, and incubated over night at 4°C with a 20-fold molar excess of SCN-Bz-DTPA (Macrocyclics, Ltd., Dallas, TX, USA). The compound was purified from unconjugated precursors by ultrafiltration and resuspended in 0.1 M ammonium acetate, pH 6.

For *in vivo* imaging, the required volume (1mg in 1mL) of the conjugated compound was incubated with 150 MBq In-111 chloride (Perkin Elmer, London, UK) for 1h at room temperature and subsequently purified from free In-111 and transferred to PBS using a PD-10 desalting column (GE Healthcare UK Ltd, Little Chalfont, UK). The purity of the labeled compound was assessed using HPLC. In-111 labeled aS100A9-DTPA was injected in amounts, corresponding to around 10 MBq per animal.

The rabbit-derived control antibody of irrelevant specificity was labeled accordingly.

### *In vivo* SPECT imaging

All *in vivo* imaging experiments were conducted on a dedicated small animal SPECT system (NanoSPECT/CT; Mediso Medical Imaging Systems, Budapest, Hungary), calibrated for In-111 emitted β-radiation. Tracers were applied in amounts, corresponding to 10 MBq/animal intravenously into the tail vein 24h before *in vivo* imaging. The mean scan time was about 60min with an initial morphologic whole body spiral CT and a consecutive whole body SPECT scan with a frame time of 60s.

Animals were held under Isoflurane inhalation anaesthesia (2% Isoflurane in air) for the whole scan time.

Data were reconstructed and analysed using in built VivoQuant Software and presented as percentage of the injected dose (%ID) per selected region of interest (ROI). For individual organ analysis, 3D ROIs were drawn on the anatomic CT images. The ROI size was identical for all parallel experiments. Mean values and standard deviation were calculated for %ID. After *in vivo* imaging, mice were either kept for longitudinal follow up examinations or sacrificed for tissue collection.

### FACS

All incubation steps were carried out at 4°C. Cells were stained with live dead (Violet Blue- or Yellow-, Invitrogen) dye in PBS for 20min, incubated with F_c_γ blocking solution in MACS buffer (Miltenyi) for 15 min. Cells were incubated for 30min with different combinations of fluorochrome-conjugated antibodies to membrane markers and then fixed/permeabilised. Intracellular staining for Foxp3 or S100A8 was carried out for 30min. Stained cells were acquired in a LSR Fortessa (BD, Oxford UK) and analysed with FlowJoSoftware (Oregon, USA). Details of antibodies used are shown in the supplementary information.

### Statistical Analysis

All data are presented as the mean ± standard deviation. Non-parametric Mann-Whitney U test or one-way ANOVA with Bonferroni post-test were used for statistical analyses, where applicable. Correlations were calculated using non-linear regression analyses. *p* values of <0.05 were considered statistically significant.

## Supplementary Material

Supplementary figures.
