# Supplementary material for: Visualization of Tumor-Immune Interaction - Target-Specific Imaging of S100A8/A9 Reveals Pre-Metastatic Niche Establishment
Source: Theranostics. 2017 Jun 15;7(9):2392–401. doi: 10.7150/thno.17138 (PMC5525744; doi:10.7150/thno.17138)
Supplement: Supplementary file 1 — Supplementary figures. [file thnov07p2392s1.pdf]

Supplementary Figure 1. Expression of surface markers defining immature myeloid cells in tumor-bearing mice.

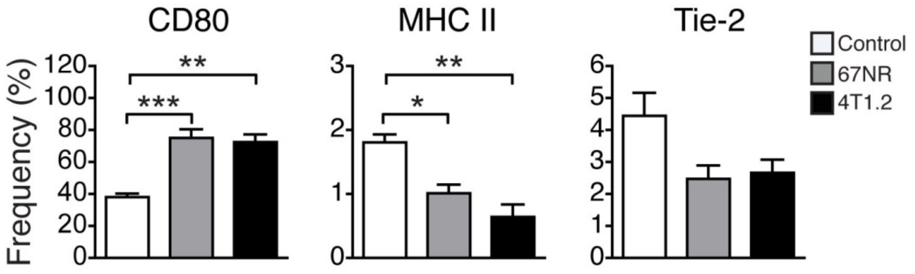

Supplementary Figure 1. Expression of surface markers defining immature myeloid cells in tumor-bearing mice. The bar graphs show mean frequency of $CD80^+$, MHC II^+, and Tie-2^+ cells gated within live $CD3^-CD19^-$ cells (see Figure 1). Non tumor-bearing control mice (n=6), 67NR (n=8) and 4T1.2 (n=8) tumor-bearing mice samples were analysed 14d after tumor induction in three independent experiments. Mean ± SE: *** $p<0.005$, **$p<0.01$, *$p<0.05$.

Supplementary Figure 1

Supplementary Figure 2. Detection of immune cell populations by flow cytometry.

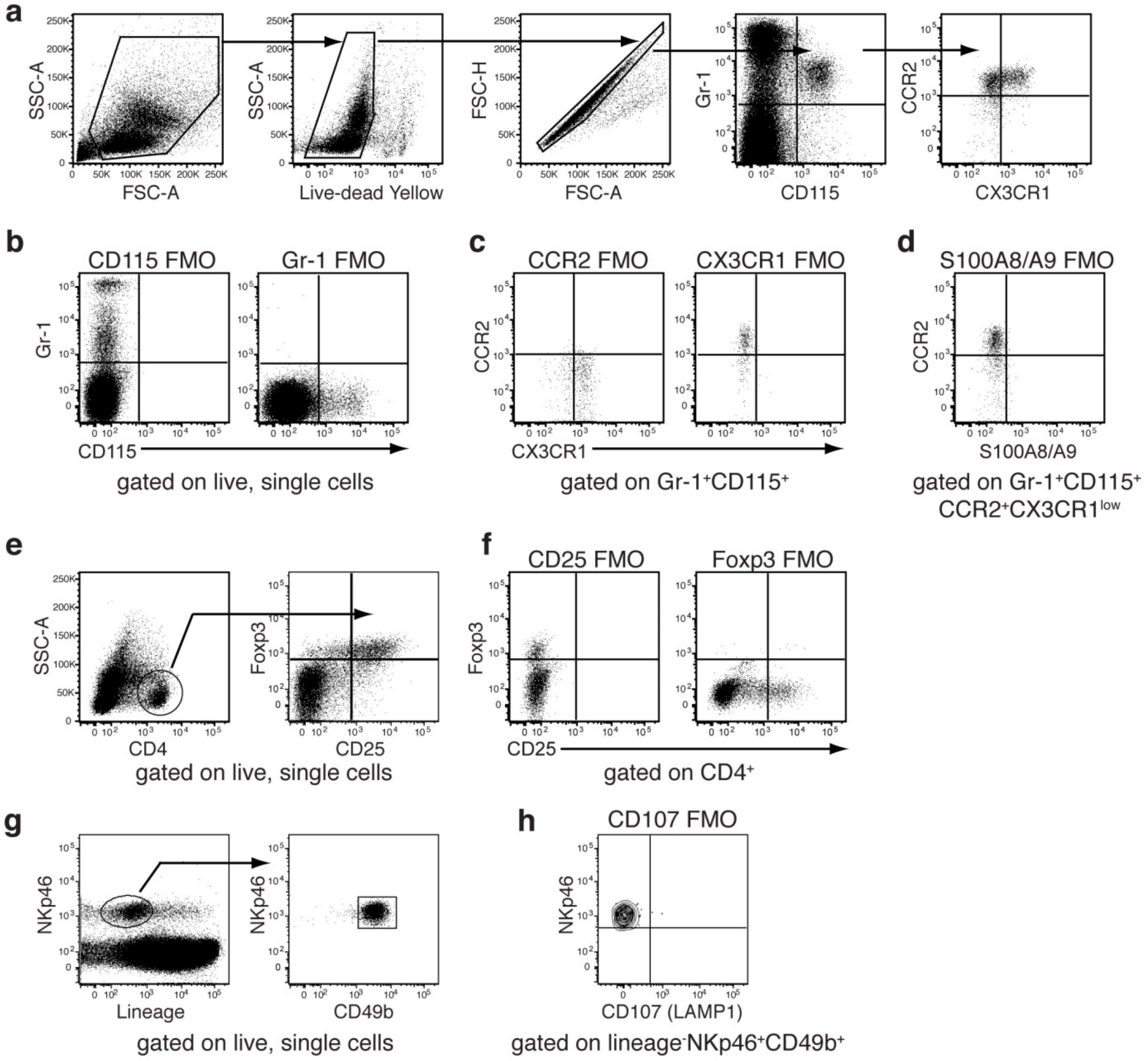

Supplementary Figure 2. Detection of immune cell populations by flow cytometry. (a) Gating strategy for detection of monocytes with Gr-1, CD115, CCR2 and CX3CR1. Sequential gating to define live (Yellow Dead-), single-cell populations (FSC-A vs. FSC-H plot) was applied. (b) Gates were set according to fluorescence-minus one (FMO) controls for Gr-1 and CD115, and (c) CCR2 and CX3CR1. (d) FMO control for intracellular staining with S100A8/A9. (e) Gating strategy for detection of Tregs with CD4, CD25 and Foxp3. (f) FMO controls for detection of Tregs. (g) NK cells were identified as lineage$^-$ (CD3, CD5, CD8, CD19, Ter119, CD115, Ly6G) and NKp46$^+$CD49b$^+$. (h) FMO control for staining with CD107 (LAMP1).

Supplementary Figure 2

Supplementary Figure 3. Pro-inflammatory monocytes in primary tumors and peripheral blood.

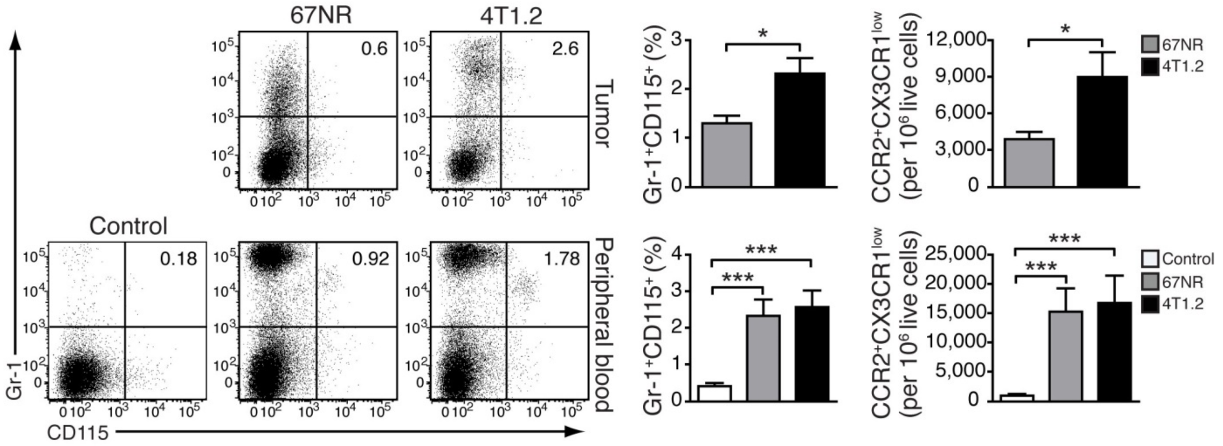

Supplementary Figure 3. FACS gating strategy for identification of pro-inflammatory monocytes. Splenocytes were stained with Yellow Dead cell stain (Live-dead) and antibodies to Gr-1, CD115, CCR2 and CX3CR1 and analysed in a Fortessa flow cytometer. Sequential gating to define live, Yellow Dead$^-$, single-cell population (FSC-A vs. FSC-H plot) was applied. Monocyte populations were defined by sequential gating in Gr-1 vs. CD115 and CCR2 vs. CX3CR1 plots. Setting of gates was according with fluorescence-minus one (FMO) controls for every fluorochrome as indicated.

Supplementary Figure 3

Supplementary Figure 4. The differentiation of NK cells is not affected in tumor-bearing mice.

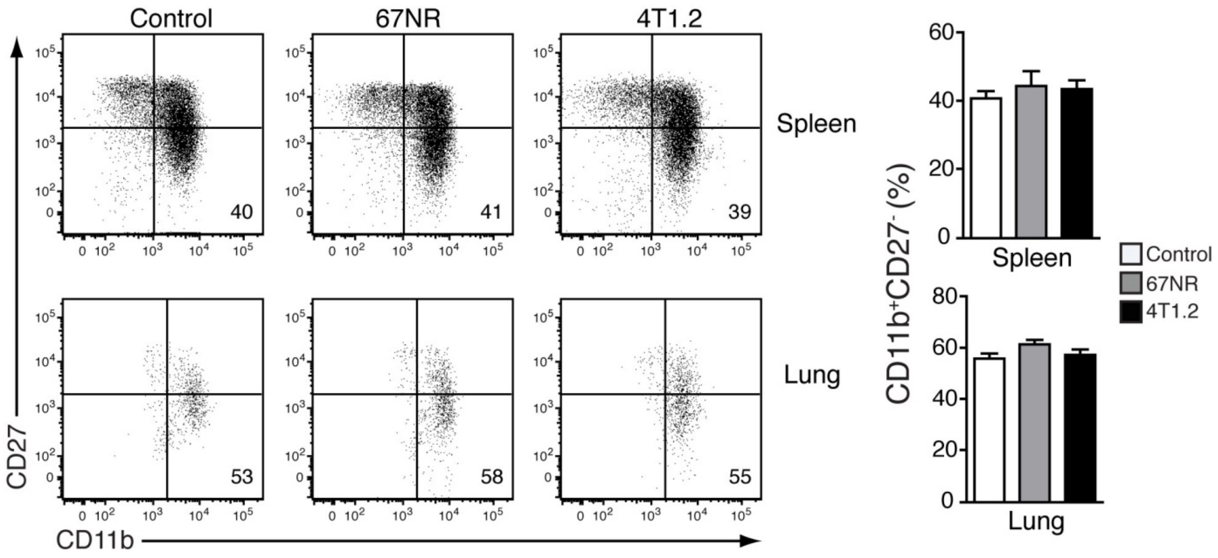

Supplementary Figure 4. The differentiation of NK cells is not affected in tumor-bearing mice. Representative plots of NK cell populations defined by the expression of CD11b and CD27. The plots show the frequency of mature NK cells ($CD11b^{+}CD27^{-}$) in spleens (top panels) and lungs (bottom panels) of tumor-bearing and control mice. The bar graphs indicate the cumulative data from two independent experiments: non tumor-bearing control mice (n=5), 67NR (n=6) and 4T1.2 (n=6) tumor-bearing mice.

Supplementary Figure 4

Supplementary Figure 5: Expression of CCL2 in lung tissue of tumour-bearing mice.

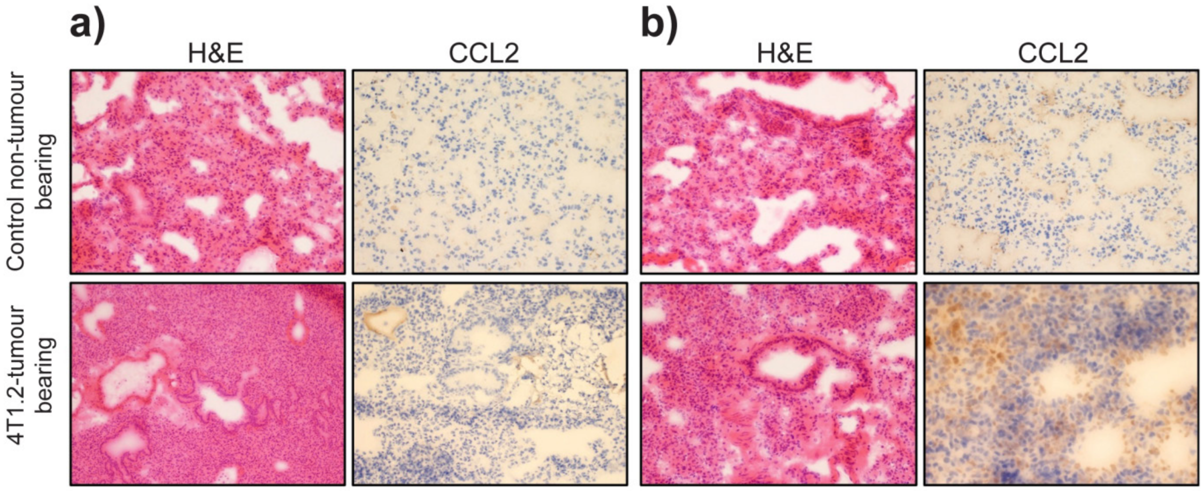

Supplementary Figure 5. Increased frequency of CCL2 positive cells in lungs of 4T1.2 tumour-bearing mice. The images show hematoxilin and eosin (H&E) staining and immunohistochemistry for CCL2 in frozen lung sections from non-tumor bearing control and 4T1.2 tumour-bearing mice at day 10 (a) and day 21 (b) upon tumour induction. This is an image of a representative experiment where groups of three mice were analysed. We found no CCL2-positive cells in the lung tissue of non-tumour bearing mice, while in lung tissue of 4T1.2-tumour-bearing mice, 5-10% of the total cellularity were positive for CCL2. CCL2-positive clusters co-localised with infiltrating immune cells. The scoring was done blindly and independently by two pathologists.

Supplementary Figure 5
